# Performance and Accuracy of the Shifted Laser Surface Texturing Method

**DOI:** 10.3390/mi11050520

**Published:** 2020-05-20

**Authors:** Jiří Martan, Denys Moskal, Ladislav Smeták, Milan Honner

**Affiliations:** New Technologies Research Centre (NTC), University of West Bohemia, Univerzitni 8, 306 14 Pilsen, Czech Republic; moskal@ntc.zcu.cz (D.M.); smetak@ntc.zcu.cz (L.S.); honner@ntc.zcu.cz (M.H.)

**Keywords:** laser micromachining, high precision, high productivity and speed, scanning strategy, ultrashort pulse laser, heat accumulation

## Abstract

A shifted laser surface texturing method (sLST) was developed for the improvement of the production speed of functional surface textures to enable their industrial applicability. This paper compares the shifted method to classic methods using a practical texturing example, with a focus on delivering the highest processing speed. The accuracy of the texture is assessed by size and circularity measurements with the use of LabIR paint and by a depth profile measurement using a contact surface profiler. The heat accumulation temperature increase and laser usage efficiency were also calculated. The classic methods (path filling and hatch) performed well (deviation ≤ 5%) up to a certain scanning speed (0.15 and 0.7 m/s). For the shifted method, no scanning speed limit was identified within the maximum of the system (8 m/s). The depth profile shapes showed similar deviations (6% to 10%) for all methods. The shifted method in its burst variant achieved the highest processing speed (11 times faster, 146 mm^2^/min). The shifted method in its path filling variant achieved the highest processing efficiency per needed laser power (64 mm^2^/(min·W)), lowest heat accumulation temperature increase (3 K) and highest laser usage efficiency (99%). The advantages of the combination of the shifted method with GHz burst machining and the multispot approach were described.

## 1. Introduction

Laser surface texturing (LST) is a technique for manufacturing micro- and nano-structures on surfaces. The textures produced often consist of periodically repeating structures (objects). LST uses pulsed lasers for the removing or moving of material by ablation or melting processes and scanning optical systems or translation and rotation stages for the movement of the laser beam on the workpiece on predefined trajectories to form the desired surface structure [1,2]. When using ultrafast lasers, LST has almost no heat-affected zone and can be applied to transparent materials as well [3].

LST was applied in different applications for the enhancement of surface properties. Braun et al. [4] reported a significant decrease in the friction coefficient in mixed lubrication by a laser-induced dimple surface texture. Vorobyev and Guo [5] produced parallel microgrooves covered by extensive nanostructures on Pt, brass and Ti that had dramatically enhanced optical absorption, super-hydrophobicity and a self-cleaning effect. Dumas et al. [6] textured titanium alloy for medical implants and found that nano-ripples favoured the osteoblastic commitment and the combination of micro-dimples with nano-ripples enhanced the osteogenic potential. Pardal et al. [7] investigated the joining (welding) of dissimilar metals (steel and aluminium) with the aim of increasing the strength of the joint by the texturing of the steel part. Different textures were tested (dimples, parallel lines, spirals and columns) with different laser welding parameters and up to a 25% enhancement was obtained. Kromer et al. [8] textured the substrate with inclined holes before a thermal spray deposition of coatings and obtained an important increase in the coating–substrate adhesion. Kümmel et al. [9] studied the influence of texture (dimples, parallel and perpendicular grooves) on the rake face of a cutting tool on the wear and build-up edge during the dry machining of steel. A decrease in tool wear and stabilisation of the build-up edge were observed using a dimple surface texture.

The limitations of current LST methods to achieve higher processing speeds are as follows: (1) heat accumulation and oxidation, (2) plasma and particle shielding effects and (3) precision at high scanning speeds. Heat accumulation (1) is a temperature increase caused by residual heat accumulated in the material from a sequence of laser pulses applied over a short period of time. The high temperature then causes effects such as oxidation and material degradation. Schonlau et al. [10] showed the experimental results of laser micromachining, indicating a degradation of the material after a significant pulse to pulse and re-run heat accumulation. Bauer et al. [11] performed experiments and numerical simulations of ultrashort pulsed laser ablation with a high-power laser. They found that there exists a threshold temperature of the material during processing above which the ablation quality decreases. The tests were done with different laser scanning speeds. Weber et al. [12] prepared an analytical model of laser multi-pulse processing of material which revealed the basic mechanisms of heat accumulation. The model agrees well with the presented experiments. The maximum average power for a good quality of machining can be predicted for a given pulse repetition rate.

Plasma and particle shielding effects (2) are the attenuation or reflection of the laser pulse by plasma and ablated material produced by the laser pulse or for high repetition rates by the previous laser pulse. Mao et al. [13] observed different depths and shapes of craters formed by picosecond laser ablation in various gas mediums and its pressures. The data correlate with a model that describes gas ionisation. The formation of plasma reduces the mass ablation and modifies the crater shape. König et al. [14] studied the transmission of a laser-induced plasma in the nanosecond time range and observed a significant reduction down to 5% at 1–10 ns and 35% at 100–300 ns. At 1 µs, the transmissivity increased to 90%. Bulgakova et al. [15] studied various effects of plasma during the laser–material interaction and have found that in some cases, plasma attenuates the laser beam reaching surface but in other cases, the good coupling between the plasma and material enhances the ablation.

Laser spot positioning with high accuracy is difficult at a high scanning speed (3). This is important mainly for complex or small objects of the texture. Further, large amounts of data need to be processed in a short time. Moskal et al. [16] tested surface laser texturing by round dimples using different methods and have found that the high-speed precise processing of small scale dimples is difficult. At higher speeds, the scanning is not precise and at a lower speed, the heat accumulation is too high.

Several proposals are described in the literature for solving these problems and for enabling a scale-up in the production of laser textured surfaces: (a) Ultra-high-speed laser beam scanning systems. Schille et al. [17] developed a polygon scanner for speeds up to 800 m/s and performed ablation experiments on different materials with a high-power laser in continuous machining (no small objects). Romer and Bechtold [18] described electro- and acousto-optic scanners and their advantages and disadvantages—high angular deflection velocities, but small deflection angles; (b) Arrays of microlenses. Li et al. [19] have studied the use of a self-assembled particle lens array with a near-field enhancement effect to write millions of nano-sized user-defined features simultaneously; (c) Multibeam interference. Burrow and Gaylord [20] described in a review the possibilities of multibeam interference for different applications. One-, two-, and three-dimensional periodic optical-intensity distributions can be generated at the micro- and nano-scale over a large length/area/volume. High aspect ratio periodic micro-structures were produced by picosecond direct laser interference patterning [21]. Three-level multi-scaled patterns containing laser-induced periodic surface structures (LIPPS) were produced on stainless steel; (d) Multispot processing. Amako and Fujii [22] reported the use of diffractive beam splitting and a Fourier transform lens during the deep-drilling of silicon using a nanosecond pulse laser split to 13, 45 or 180 spots. Silvennoinen et al. [23] described parallel processing using a spatial light modulator with computer-generated holograms. Up to 576 spots were used to produce microfeatures in sizes down to 1 µm; (e) Interlaced texturing mode. Neuenschwander et al. [24] investigated different strategies for high-throughput surface structuring and calculated the surface temperatures at the time of the next pulse. An interlaced texturing mode was proposed; (f) Optimizing the efficiency of ablation [25]; (g) Using multiple scan heads in a laser system. These methods enabled advances in some aspects of the problems mentioned above (e.g., the heat accumulation effect) for certain cases, but a general and versatile solution including the formation of precision-shaped objects at a high scanning speed and a simple computational description of large arrays of micro-objects is not available yet.

The shifted laser surface texturing method (sLST) was developed to solve the mentioned limitations. It is a method based on high-speed laser beam scanning on a raster of straight lines with a constant speed and pulse repetition rate and small shifts of the lines’ positions before the next scanning. The sLST was used to manufacture various textures with simple or complex microscale geometries, e.g., dimples [26], square columns [27], circular columns and donut holes [28,29], sloped holes [29]. The method promises high processing speeds with a high accuracy of textures produced at high scanning speeds [27]. The purpose of this paper is to present a quantitative assessment of accuracy and performance of the method, in order to verify whether the method meets the requirements and solves the mentioned limitations. This is done by an investigation of the one-layer texturing precision for different scanning speeds and by a depth profile shape accuracy assessment of the complete texture depth (multi-layer process) for various texturing methods at their highest possible scanning speeds. The experiments focused on obtaining the highest processing speed for each method while maintaining the precision and surface quality of the resulting texture. The heat accumulation temperature increase and efficiency of the laser usage are also calculated for each method.

## 2. Laser Surface Texturing (LST) Methods

LST methods are based on different scanning strategies of the laser beam. The material is removed by ablation in ultrashort laser pulses and the pulses can be placed in different sequences and orders. Certain methods use straight lines (raster scanning), but other methods use curved lines (vector scanning). The combination of the final number of laser pulses on different locations forms the shape of the surface structure.

**Figure 1:**
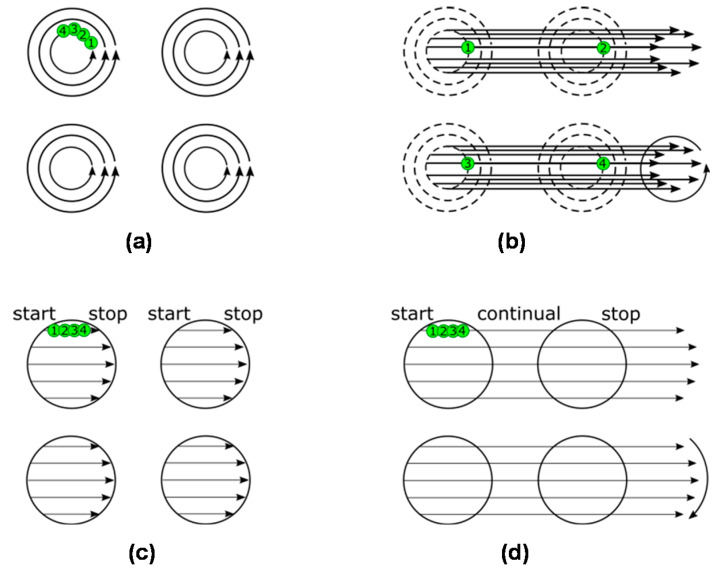
Schematic representation of the laser surface texturing (LST) methods: (**a**) classic path filling, (**b**) shifted path filling, (**c**) classic hatch and (**d**) shifted burst. Green dots represent laser pulses. Numbers describe the order of laser processing. Black arrows indicate the movement of the laser beam during pulsing. Curved arrows for shifted methods represent the shifting of the raster during the process.

In this paper, four texturing methods are compared. Two currently used methods are presented first: (1) path filling (referred to as the “classic path”) and (2) hatch over all objects (referred to as the “classic hatch”). The first method (Figure 1a) is based on laser beam scanning on curved lines (paths) and filling the objects by laser pulses on an offset hatch. The method is sometimes also called the “scaling strategy”. For circular objects, the laser will be scanned on concentric circles. All paths are processed in one object before passing to another object. Laser beam scanning on the curved trajectory is limited by the need of an acceleration most of the time, so for high-precision processing, only small speeds can be used. The second method (Figure 1c) is based on scanning straight lines in certain distances. The lines start and end on the borders of the objects. Straight lines can be scanned easily at a high speed (no acceleration required), but the laser beam switching is difficult to be precisely done at high speeds. Moreover, the commercial software used does not scan the line continuously over all objects, but by separate short lines—one for each object. It means starting and stopping the scanning process at each object (Figure 1c). Both of these methods can be also limited by heat accumulation and plasma shielding at a high repetition rate of laser pulses, as a high number of pulses are placed over a small area in a short time.

The other two texturing methods in comparison are two variants of the shifted laser surface texturing method (shifted LST or sLST) [27,28,30]: (3) sLST using one laser pulse per laser spot (denoted “shifted path”) and (4) sLST using a burst of pulses per one laser spot (denoted “shifted burst”). In sLST, laser pulses are rapidly distributed to the entire processed surface by fast scanning on straight lines. In the shifted path method, only one laser pulse is applied to each object (Figure 1b). The scanning is done on a raster of straight lines and the laser pulsing is switched-on continuously during the processing of whole lines. In each next repetition, the raster is slightly shifted on the surface. By a combination of separate laser pulses obtained from different raster positions, an array of objects is formed on the surface. The sequence of raster shifts forms the shape of each object. The heat accumulation temperature increase is very low because the next laser pulse comes to the same object after the scanning of the whole raster. The plasma shielding effect is also low because the following laser pulse is placed in a separate object at a relatively long distance, not covered by the plasma of the previous pulse. The computational resources are less needed, as there is no direct description of each object in the array. Finally, precision at high scanning speeds is possible because no acceleration is necessary, and the laser pulses at a constant frequency.

The shifted burst method is shown in Figure 1d. One laser spot is composed of several laser shots (a burst of pulses). The number of pulses in the burst is constant for each spot in one line. For each object, only one line is done in each raster repetition. Then for the next repetition, the raster is shifted down and a different number of burst shots inside of one spot is set according to the width of the object at this location. For inverse objects, such as circular columns, the burst sLST method can also be used with benefit (see the examples of produced textures in Supplementary Figures S1 and S2). The depth of the texture will be the same in all places, not like for square columns done by cross-line scanning, where in the line intersections, the depth doubles. In burst sLST, heat accumulation occurs because of directly overlapping pulses, but it is moderate and it can help the process efficiency and stability if the material temperature affected by the heat accumulation is kept under the threshold temperature of material degradation [28]. Another advantage, in comparison with the classic hatch over all objects method, is that the scanning is run continuously at a constant speed. For the classic hatch method, the scanning is done separately for each object and the laser has to be synchronized (switched on and off) for each such separate scanning line.

The sLST method is ideal for the production of textures with periodical shapes and structures (array of objects, e.g., as in Supplementary Figure S3). It can be used also for larger continuous objects (e.g., grooves), but it is not suitable for irregular, random and not periodic structures.

## 3. Experiment and Evaluation Methods

The performance of different LST methods was evaluated on a selected surface texture. The experiments focused on obtaining the highest processing speed for each method while preserving the precision and surface quality of the resulting texture. The classical methods chosen for comparison were (1) classic path (concentric circles) and (2) classic hatch. The shifted LST method was evaluated in two variants: (3) shifted path (shifts on concentric circles) and (4) shifted burst. The resulting texture geometry was assessed in two ways: (A) size and circularity of the dimples from a normal view and (B) depth profile shape of the dimples.

The experiments were conducted using a picosecond laser system with a galvanometer scanning head. The laser (PX25-2-G, EdgeWave, Würselen, Germany) has a pulse duration of 10 ps, a wavelength of 532 nm, a maximum pulse energy of 50 µJ and an average power of 14 W. The galvanometer scanner (intelliSCAN III 14, SCANLAB, Puchheim, Germany) has a 255 mm focal length f-theta objective. In this configuration, the spot diameter is 23 µm and the highest laser beam scanning speed is 8 m/s. This speed is not sufficient for a full use of the laser; therefore, only certain laser pulses were used by the application of an external trigger. Full use would be possible with ultrafast scanning equipment, such as the polygon scan head. The scan head was controlled by an RTC5 control card (SCANLAB). Texturing by the classical LST methods was done by the Laser Desk software (SCANLAB) and texturing by the shifted LST methods by the Laser Control software application developed by the authors.

The texture to be used to compare the methods was composed of dimples with a diameter of 80 µm and a depth of 6.5 µm at distances of 200 µm. This type of texture is applicable, for example, for the enhancement of tribological properties in sliding bearings [26]. The processed material was cold-rolled AISI 304 stainless steel. Laser pulses with a pulse energy of 10–12 µJ were used. They were overlapped with a distance of 7 μm in the lines and the distance between the lines was 10 μm. This means an overlap of 70% in the lines. The overlap was the same for both the classic and sLST methods, only with the pulses occurring at different times. The scanning speed ranged during the experiments from 0.02 to 8 m/s and the pulse repetition rate was adjusted from 3 to 1.2 MHz to obtain the chosen overlap.

**Figure 2:**
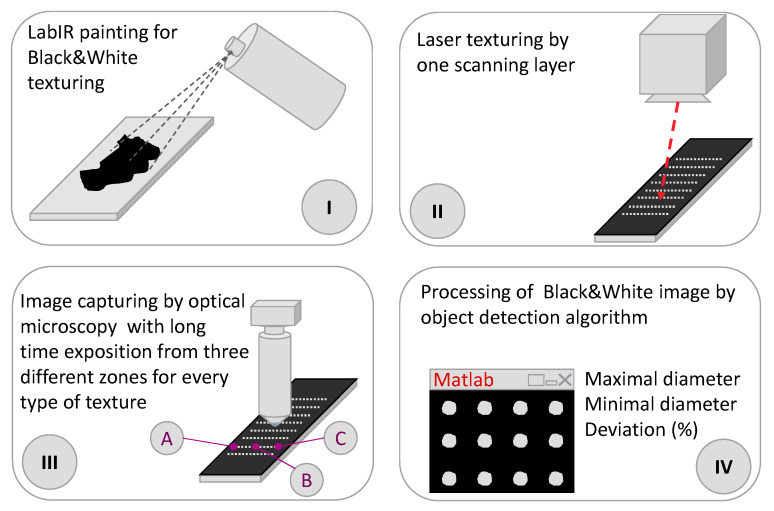
Sketch of the methodology for the determination of the surface dimple diameter by using LabIR paint, an optical microscope and software analysis.

The first assessment of the precision of the texture geometry was the size and circularity of the dimples from a normal view. This was done by the procedure shown in Figure 2. The sample surface was first covered by LabIR high-emissivity paint (HERP-LT-MWIR-BK-11, University of West Bohemia, Pilsen, Czech Republic). Subsequently, only one layer (one repetition) of the laser surface texturing was applied. The paint was removed in the areas processed with the laser. In the third step, the samples were photographed by an optical microscope (KH-7700, Hirox, Tokyo, Japan) with a long exposure and a high-contrast image was obtained for an easy and precise software analysis of the dimple size. In the last step, the images were analyzed by a MATLAB program (MathWorks, Natick, MA, USA).

**Figure 3:**
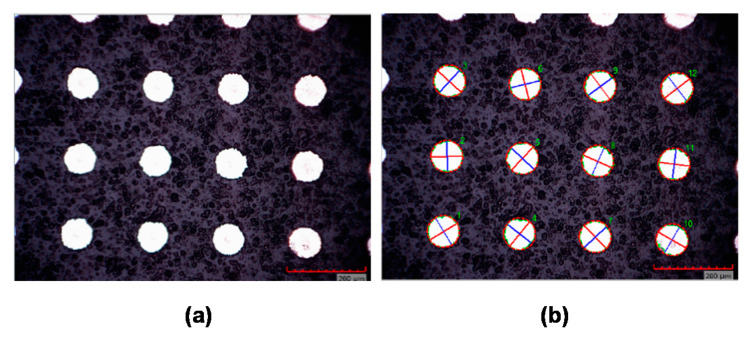
Example of the analysis by a Matlab function: (**a**) high contrast image of dimples from an optical microscope, (**b**) analyzed microscope image with shown fitted ellipses and their axes (red—major axis, blue—minor axis).

The analysis of the images in MATLAB was based on the following steps: (1) conversion of the grayscale data to binary data (intensity image to black and white), (2) detection of the borders of the textured objects, (3) deletion of wrong objects (for example on edges), (4) detection of the center for every object (ellipse), (5) detection of the maximal and minimal diameter from every object (major and minor axes of ellipse), and (6) statistical analysis of the differences between the goal diameter and the detected diameters. An example of the performed analysis is shown in Figure 3.

The statistical analysis was done together for three places on a sample for each texture—the center and close to both ends. For each place, one microscope image was analyzed containing about 12 objects. Together from the three places, *N* objects were identified and *2N* diameters were determined (major and minor axes). Absolute deviations were calculated as an absolute value of the difference in the detected and goal diameters. Relative deviations were calculated as an absolute deviation divided by the goal diameter. Finally, the mean value and standard deviation of the relative deviations were calculated from the *2N* values.

**Figure 4:**
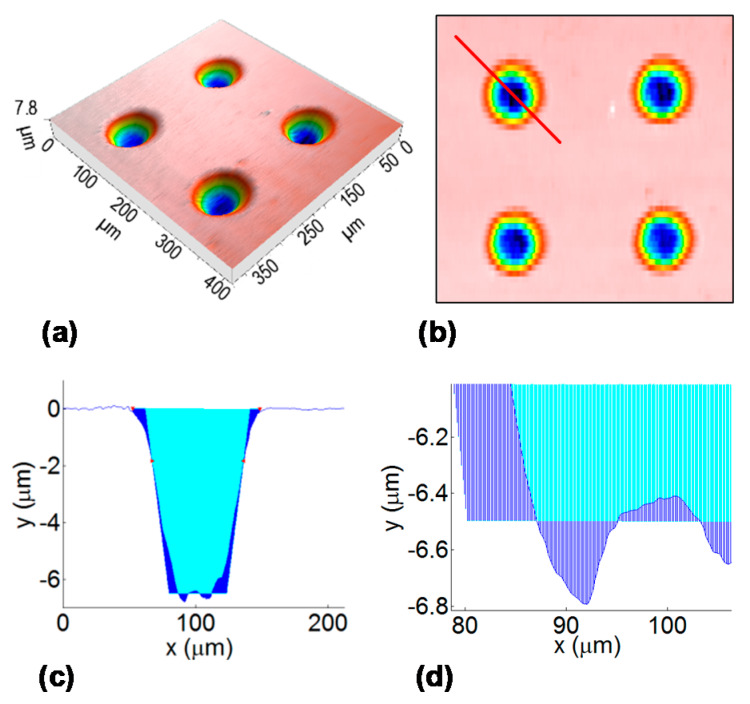
Sketch of the methodology for the determination of the dimple depth profile: (**a**) measured 3D profile, (**b**) top view of the 3D profile with a diagonal line, (**c**) measured linear depth profile in the diagonal compared with the goal profile (blue area represents deviation), (**d**) detail of the bottom part.

The second assessment of the precision of the texture geometry was the depth profile shape of the dimples. This was done by the procedure shown in Figure 4. The produced full texture (using a multi-layer process) was analyzed by a contact surface profiler (P-6 Profiler, KLA-Tencor, Milpitas, CA, USA) in the parallel lines with a length of 400 μm and a separation distance of 8 μm. The profiler tip angle was 60° and its radius was 2 μm, and it used a load of 1 mg and a speed of 50 μm/s. The profiler lines were scanned perpendicular to the laser scanning direction. The 3D surface profile was reconstructed from the linear profiles (Mountains Map Imaging Topography software, Digital Surf, Besançon, France). The linear depth profile of each dimple was obtained from the 3D profile by placing the line at a 45° inclination (diagonal). This inclination was used to include data and errors from both the laser scanning and profiler scanning. The obtained linear depth profile was then compared to the goal depth profile. The goal profile has a shape of a trapezoid, with a taper angle of 70° and a depth of 6.5 µm. The goal tapper angle was calculated for the goal depth using a theoretical ablation profile equation [31,32] and ablation threshold fluence [33] corrected by the incubation coefficient of 0.85 [34].

The comparison of the measured depth profile with the goal profile was conducted in the following steps: (1) levelling of the profile and detection of the center line (by dividing the profile on two equal areas: top and bottom); (2) putting a 70° slope on the walls in the center line; (3) determination of the difference in the measured and goal profiles (y direction) at each point of the x direction (horizontal); (4) summation of the absolute values of the difference multiplied by the step in the x direction; and (5) statistical analysis of the sum of differences between the measured and goal depth profiles.

The statistical analysis was done together for three places on a sample for each texture—in the center and close to both ends. For each place, one 3D profile was analyzed containing 4 objects. Together from the three places, *M* objects were identified. Absolute depth deviations were calculated as the sum of the absolute values of difference in the measured and goal depth profiles multiplied by the step in the x direction. The area of the goal depth profile was calculated in the same way as the sum of the values of the depth of the goal depth profile multiplied by the step in the x direction. Relative depth deviations were calculated as the absolute depth deviation divided by the area of the goal depth profile. Finally, the mean value and standard deviation of the relative depth deviations were calculated from the *M* values.

The processing speed (mm^2^/min) was calculated as the processed area divided by the duration of the laser process. There were two sizes of the processed area: 2 × 10 mm^2^ and 2.4 × 170 mm^2^. The longer size was in the direction of the laser beam scanning (for the shifted and hatch methods). In these two variants, 500 and 10,200 objects (dimples) were produced on the surface. The duration of the laser process was determined by two ways: measured experimentally during processing and as predicted by the scanning software.

## 4. Results and Discussion

In this section, the results of the laser surface texturing are shown and discussed.

### 4.1. Size and Circularity of Dimples

**Figure 5:**
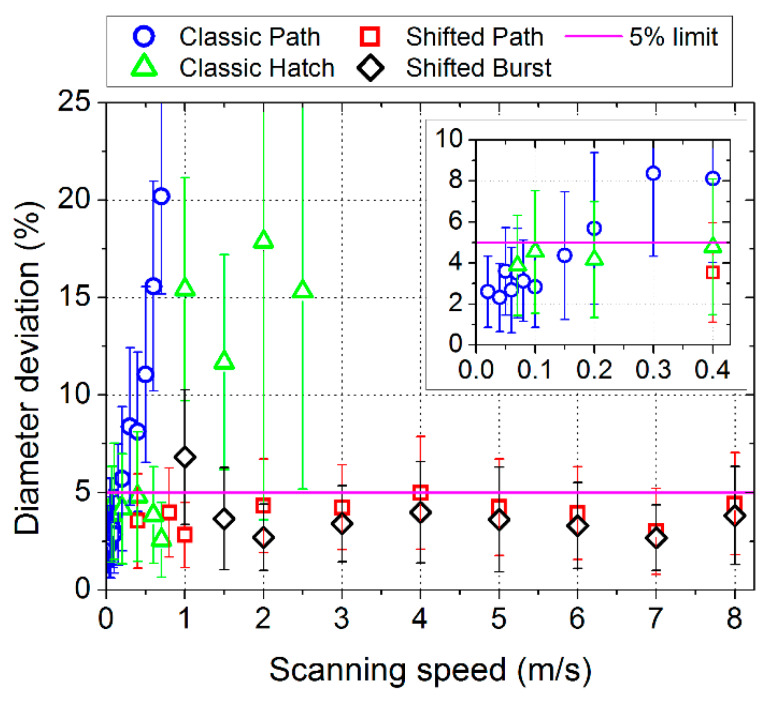
Measured relative deviation of the surface dimple diameter depending on the laser beam speed for different laser texturing methods (scanning strategies).

The results of the size and circularity of the dimples depending on the laser beam scanning speed are shown in Figure 5. The diameter (size and circularity) deviation limit of 5% was chosen to assess the texture as having acceptable precision (precision limit). It can be seen that for the classical methods, a certain scanning speed threshold exists. Below this value, the precision of the texturing is acceptable. For higher speeds, the deviation in the dimple diameter increases significantly and the results are not acceptable. The classic path filling method has acceptable precision in scanning speeds up to 0.15 m/s. The deviation value linearly increases and at 0.7 m/s, the deviation is higher than 20%. The size and circularity of the dimples has relatively good repeatability also at higher speeds (although the deviation is high).

**Figure 6:**
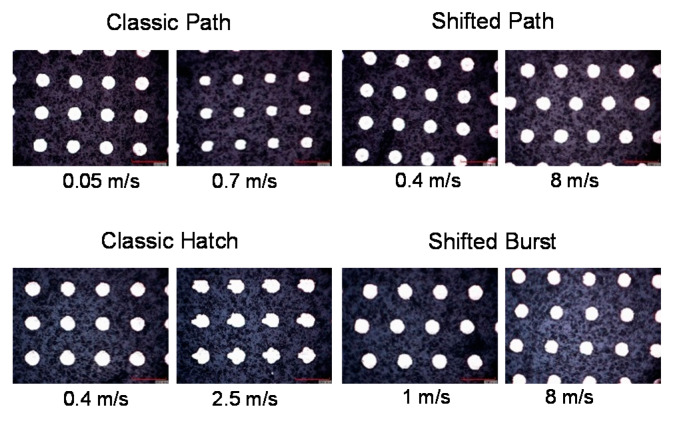
Microscope pictures of the surface dimple shapes for different texturing methods (scanning strategies) and laser beam speeds.

The classic hatch method gives results with acceptable precision up to a scanning speed of 0.7 m/s. For higher speeds, the shape of the dimples becomes irregular, as shown also in Figure 6. At a speed of 1 m/s, the average deviation exceeds 15%. For higher speeds, the diameter deviation is similar, while the standard deviation is extremely high—the result is not repeatable for different parts of the textured area. The irregularity of the shape is caused by the timing of switching the laser beam on and off, which is difficult at high speeds.

For the shifted LST method, the results have acceptable precision for a wide range of scanning speeds for both variants—path filling and burst (Figure 5 and Figure 6). For the shifted path method, the diameter deviation is lower or equal to 5% for all tested laser beam scanning speeds (0.4–8 m/s). For the shifted burst method, the diameter deviation is highest at low speed and becomes smaller at higher speeds. At 1 m/s, the deviation is 6.8%, which is not acceptable, but for other tested speeds, it is lower than for the shifted path method. Both variants of the shifted LST method are intended to be used at high speeds; therefore, the higher deviation at low speed for the burst variant should not pose any problem.

In order to increase the processing speed, the scanning speed can be increased even higher: up to 50 m/s with fast galvanometer scan heads or up to 1 km/s with, for example, polygon scan heads, and the presented shifted method should perform well also at these high speeds. The applied laser power in the shifted methods is limited by the scanning speed because the frequency of the passed laser pulses is limited by the scanning speed for the equidistant laser spot distribution in the raster. The increase in the laser beam scanning speed gives the possibility to increase the frequency of the applied laser pulses and as a result, the processing speed will be higher. The shifted method needs synchronization only at the beginning of the scanning line. Then, during scanning it needs only a constant scanning speed and constant repetition rate of the laser pulses (shifted path), or an external trigger opening at a constant repetition rate (shifted burst). All these issues can be solved with nowadays or near future equipment.

Due to the asynchronous manner of the laser pulses’ positioning in the shifted method, the micro-objects in the rows don’t have guaranteed absolute positions (Figure 6, shifted path and shifted burst methods). On the other hand, the relative positions keep constant. The scanning is done in both directions in order not to lose time. Therefore, each odd row of objects starts on the left side and the even row at the right side. The correction of the micro-objects’ positions in the shifted methods can be solved by replacing every odd line start position on the new position of several microns to the left or right direction. It does not affect the processing speed and should be optimized individually for every type of texturing if it needs to have a prescribed distribution of the micro-objects on the textured surface.

### 4.2. Depth Profile of Dimples

**Figure 7:**
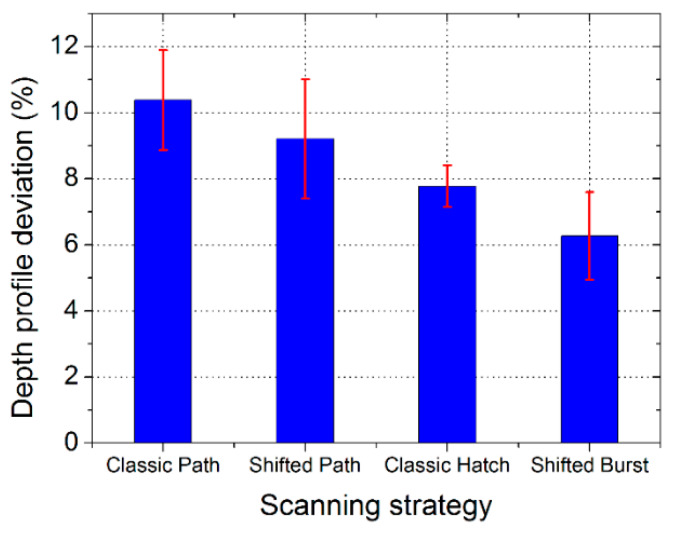
Measured relative deviation of the dimple depth profile for different laser texturing methods (scanning strategies) at their highest allowed scanning speeds.

The second assessment of the resulting texture geometry was the depth profile of the dimples. The overall results of the depth profile deviation for the different texturing methods are shown in Figure 7. The presented results are for the highest allowed laser beam scanning speed for each method with an acceptable dimple size and circularity from the normal view (previous test): 0.15 m/s for the classic path, 8 m/s for the shifted path, 0.7 m/s for the classic hatch and 8 m/s for the shifted burst method. For all methods, the depth profile deviation is lower than or around 10%, so the precision of the shifted method in both variants is comparable or even better than for the classical methods, although done at a much higher scanning speed.

**Figure 8:**
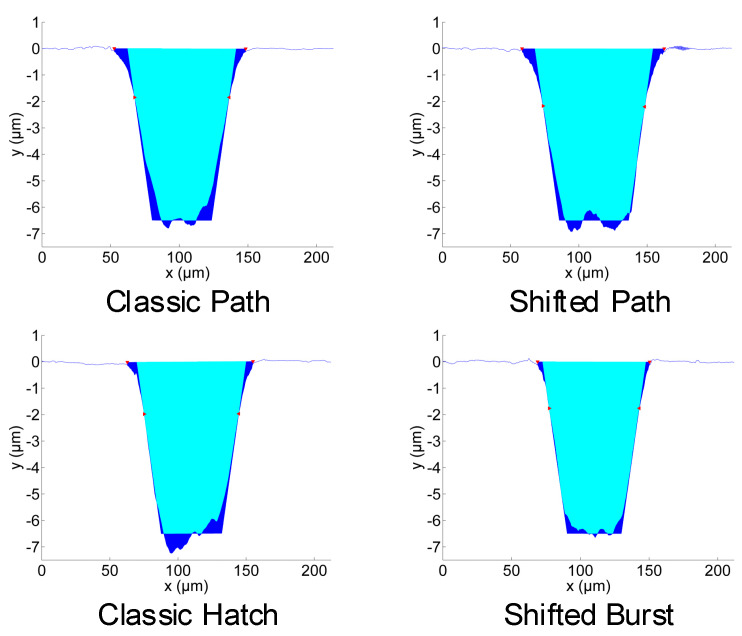
Comparison of the depth profiles of the dimple produced by different laser texturing methods (scanning strategies) at their highest allowed scanning speeds.

**Figure 9:**
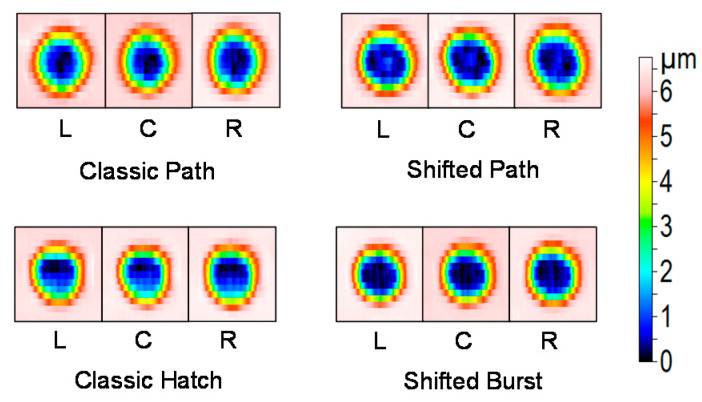
Comparison of the 3D profiles of the dimples produced by different methods (scanning strategies) at their highest allowed scanning speeds. Images are taken from different places on the sample: L—left, C—center, R—right.

Examples of the measured depth profiles and deviations from the goal depth profile are shown in Figure 8. The most significantly different shape of depth profile is for the classic hatch texturing method. The dimple bottom is not flat, it is inclined. This is also seen in the 3D profile views in Figure 9. It may be in part attributed to the heat accumulation or incubation. However, the heat accumulation is not high enough for such a high influence (Table 1). The depth change at the bottom is mainly present in the laser beam scanning direction (vertical in Figure 9). It means that the effect of previous lines is almost negligible.

For the classic path method, the central part of the dimple is deeper (with the bottom being narrower, Figure 8). This may be partly caused by the higher temperature and higher incubation effects in the center, where most laser pulses coincide in a short time. For the shifted burst method, the heat accumulation (or incubation) forms at a different time scale (fast scanning and high repetition rate) than for the classic hatch method and probably occurs immediately after several pulses and is stable during most of the line inside the dimple.

**Figure 10:**
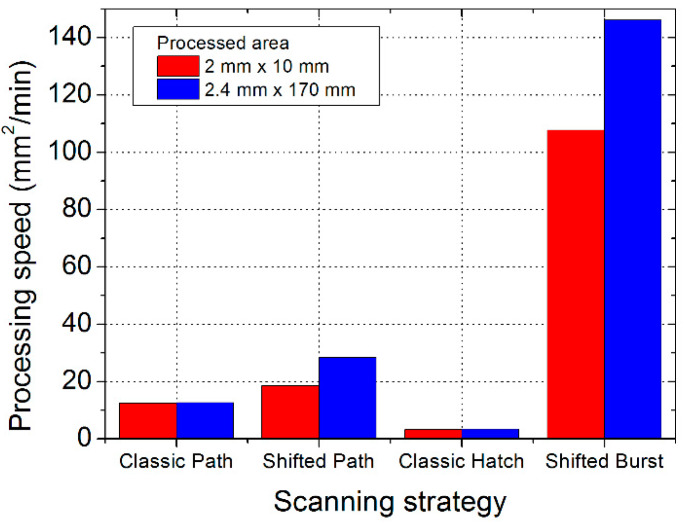
Processing speed obtained for different laser texturing methods (scanning strategies) at their highest allowed scanning speeds. The data are based on the measured processing times for two different processed areas.

The heat accumulation temperature increase (Δ*T*) is shown in Table 1. This is the temperature difference of the material surface in the short time before the next laser pulse compared with the initial temperature. It is caused by residual heat from previous laser pulses. It is calculated for all methods using the simplified semi-planar model of a pulsed-scanned source described in [28]. The heat accumulation is calculated for the straight-line scanning of the laser beam across the calculated position. Four laser pulses are mainly overlapped over one place. The heat accumulation rises after each pulse and the maximum value is taken into account. The highest temperature increase is for the shifted burst method (320 K) and lowest for the shifted path method (3 K). For the shifted path method, the result is difficult to calculate because the time is exceedingly long. This value is for the small area of 10 mm long (see Figure 10). For the big area, the temperature would be even smaller. For the classic path method, up to nine pulses overlap for the smallest circle. The heat accumulation temperature increase is expected to rise up to 150 K. However, even this temperature is probably not high enough to present a strong influence on the ablation rate. The heat accumulation for the shifted burst method (Δ*T* = 320 K) can already affect the process (increase ablation rate), but not damage the surface (Δ*T* = 582 K, 607 °C reported as a critical value [11]). On the other hand, the measured ablation rate is rather lower (Table 1). This can be caused by particle shielding possibly present in the MHz range of the repetition rates [14,35].

The number of repetitions of the texturing process for the complete depth was different for the different methods. It was set after several tests in order to obtain the required depth profile. For the classic path, it was 45 repetitions, for the shifted path 60, for the classic hatch 85 and for the shifted burst 90 repetitions (layers, Table 1). The classic path method gives a narrower dimple at the bottom (Figure 8 and Figure 9). For the classic hatch and the shifted burst methods, almost the same number of repetitions are used. The difference between these two and the path methods can be caused by the different shape of the scanning lines for the path and hatch methods and the bigger distance between the lines (10 µm) than the distance of spots in-line (7 µm).

The ablation rates obtained in this study ranged from 0.4 to 0.7 µm^3^/µJ (Table 1). The ablation rate for stainless steel in the literature were found in the range from 0.2 to 1.7 µm^3^/µJ for picosecond lasers [17,33,35,36] and up to 6 µm^3^/µJ for femtosecond lasers [33,37,38,39]. The present values are in the range found in the literature (for picoseconds lasers). They are not at the maximum values, but this was not the goal of this study. This can be caused by a higher fluence or particle shielding for higher frequencies [14,35]. The fluence used in the experiments (2.65 J/cm^2^) was higher than the most efficient fluence for stainless steel (0.69 J/cm^2^ [17]), but the comparison of the methods should be valid. The reason for using a higher fluence was that the process performed well (according to the experience) and higher processing speeds were obtained (better for application). For working at the most efficient fluence and for having higher processing speed, a higher repetition rate would be required. That would mean a higher overlap for the fixed (maximum) scanning speed. However, the minimum surface roughness is achieved with a spatial overlap of 50–75% [33]: this is why in this work a 70% overlap is used.

The ranges of the values for the classic hatch and shifted burst methods in Table 1 are given because it had not been clear how many pulses are placed inside of the dimple. The possible number of pulses is given and then the ablation rates are calculated. The number of pulses is precisely given only for the shifted path method (positions of different rasters). The other methods use scanning lines with a certain time of opening of the external gate and the number of pulses can differ by ±1 for each line. There are seven lines for a dimple for the classic hatch and shifted burst methods and three lines for the classic path method (diameters 20, 40 and 60 µm).

In Figure 9, 3D images from various places of the textured samples are shown: left, center and right. It can be seen that for each method, the process is repeatable with only small changes. Higher differences can be identified between the texturing methods. The statistical analysis of the measured depth profiles is shown in Figure 7 as error bars for the different texturing methods. The lowest difference and thus highest repeatability of the depth profiles were found for the classic hatch method.

### 4.3. Processing Speed of the Laser Texturing Methods

**Figure 11:**
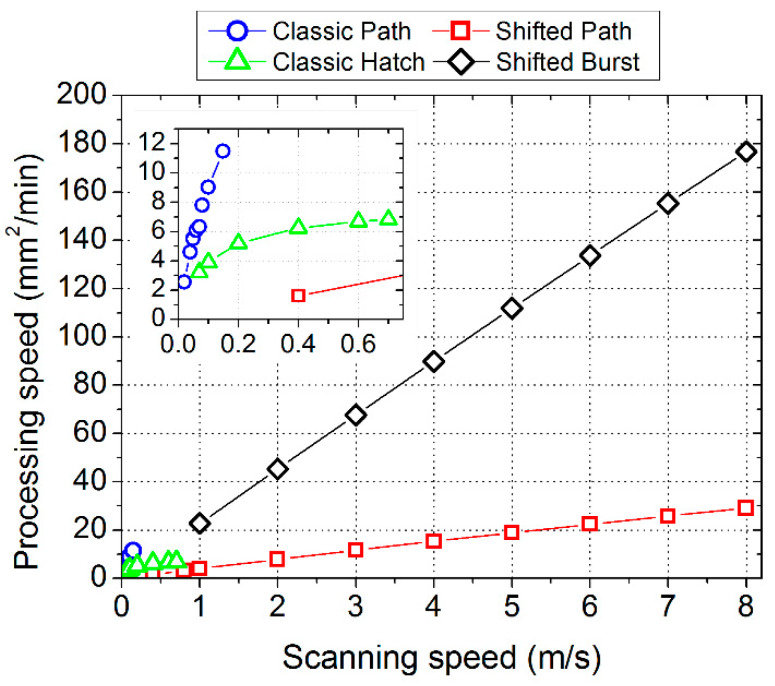
Processing speed calculated for different laser scanning speeds and for different laser texturing methods (scanning strategies). The data are based on the processing time calculated by the scanning software for the processed area of 2.4 × 170 mm^2^.

In the last section, the productivity of the different methods is compared. The obtained processing speed is shown in Figure 10 for the different texturing methods and the two processed areas. These results are based on the measured time of the laser processing of the complete dimples for the highest laser beam scanning speeds possible for the different texturing methods. The highest processing speed was found for the shifted method in the burst mode—146 mm^2^/min. The lowest processing speed was identified for the classic hatch method—3.4 mm^2^/min. This is a major difference, indicating the shifted burst method being 43 times faster. The processing speed was also calculated from the software prediction of the processing time (Figure 11). In this case, the maximum processing speed for the classic hatch method was more favourable—6.8 mm^2^/min—but still lower than for the other methods. The difference between the software prediction and the real measured production time can be caused by synchronization issues of the laser with the scanning head. It is possible that for other laser or other synchronization methods, the experimental results will be closer to the predictions. For the shifted burst method, the predicted processing speed (177 mm^2^/min) is also higher than the one obtained from the experiment. For the classic path and shifted path methods, the agreement of the predicted and measured processing speed is good.

Differences in the processing speed for the shifted method in both variants were found for the different sizes of the processed area. For the classic methods, there is almost no difference for the different processed area sizes. For the classic path method, the explanation is clear: the texture is processed one object by another (all circles in one dimple and then move to other dimple), so the processing time of the whole texture roughly equals the processing time for one object multiplied by the number of objects. For other methods, the importance of time for the setting up of the movement of the galvanometer scanning mirrors to the set speed (jump delay) and the time for the synchronization of the laser with a scanner increases. For the shifted method in both configurations, this takes place at the beginning of the scanning line (one side of the processed area). For the classic hatch method, this is done before each dimple. This is the reason why the classic hatch method has the lowest processing speed and the speed does not depend on the area size in our experiments. This loss of time at the beginning of each line (and each dimple) is limited to the classic hatch method, which looked very promising from a theoretical point of view. This explanation is also supported by the trend of the processing speed in the dependence on the scanning speed (Figure 11). The processing speed has a linear dependence on the scanning speed for the other three methods, but for the classic hatch method, it has a certain limit and the processing speed does not increase with the laser beam scanning speed any more.

The loss of time at the beginning of each line is also the explanation for the increase in the processing speed with an increase in the processing area length for the shifted method (in both variants). For the shifted path method, there are more scanning lines (one line—one pulse per object) in the process than for the shifted burst method (one line—burst of pulses per object); therefore, the effect of area size becomes more pronounced (increase of 52% compared with 36%). The dependence of the processing speed on the area size for the shifted method can be a limiting factor for its application on small areas. As such, its main application is for large areas—fully using the scanning field of the scanning system.

Finally, the productivity of the methods and their potential in the future is compared. The highest processing speed of the classic methods was experimentally observed for the classic path method (12.6 mm^2^/min) and its increase with an increase in the scanning speed is not permitted by a loss of precision. The only increase in this method would be by including hardware with a higher acceleration of the mirrors (e.g., electro-optical deflectors or acousto-optical deflectors [18]). The lowest processing speed was obtained for the classic hatch method; here, too, its increase with the increase in the scanning speed is not permitted by a loss of precision. An increase in this method would require an advance in the synchronization (hardware and software) between the laser and the scanning head and the elimination of the stopping of the scanning between the dimples. The presented shifted method solves/eliminates many of the mentioned problems (e.g., the need of high acceleration, stopping between pulses). The shifted method reached a processing speed of 28 mm^2^/min for the path variant and 146 mm^2^/min for the burst variant at the scanning speed of 8 m/s. This means a processing speed 2.2 times higher than the best classic method for the shifted LST method in the path filling variant and more than 11.6 times higher for the burst variant.

The obtained processing speed can be compared with the literature. Recently published results [21] using another promising method, direct laser interference patterning (DLIP, multibeam interference), have shown a processing speed of 7–15 mm^2^/min for ferritic stainless steel and a similar depth of texture (4–12 μm). The present results of the processing speed are comparable or even 10 times better for the case of the shifted burst method. The DLIP is an interesting method. It has advantages of the production of surface textures on a larger area simultaneously with the possibility of the production of very small surface features down to a 0.5 µm spatial period [40,41]. It has already achieved a processing speed of 0.9 m^2^/min for the surface structuring of plastics (foaming by a one-layer process) [42]. A disadvantage can be the limited variability of the surface texture—only the surface textures resulting from the interference can be produced.

Table 2 shows the laser usage efficiency for the different laser texturing methods. It is calculated by the jump time efficiency multiplied by the geometrical efficiency. The jump delay time is a sum of all jump delays in the process. For the classic hatch method, this time is exceedingly high. It constitutes half of the entire processing time. This shows one of the principal differences compared with the shifted burst method, where the jump delay time is negligible.

The geometrical efficiency is a ratio of the time when the laser is “on” during scanning to the overall time of the laser scanning (without jump delays). It is low for the classic hatch and shifted burst methods. It depends on the texture geometry. For inverse textures, such as circular columns, this efficiency would be significantly higher. On the other hand, both path methods have good values in this efficiency.

The laser usage efficiency is particularly important for the overall economical budget of the technology. A laser source with a higher average power is more expensive than a source with a lower power. If this laser power is used only at 23% (shifted burst) or 12% (classic hatch), it means that there is high investment in not paying off fast enough. From this point of view, the path methods deliver a better performance (78% and 99%). However, the classic path method is limited by the scanning speed. The shifted path method is expected to work also at a high scanning speed with the same laser usage efficiency (99%).

The high laser usage efficiency of the shifted path method can be a big advantage for a combination of this method with a GHz burst laser ablation. The GHz burst femtosecond laser machining has shown very promising results in the ablation rate and ablation cooling [43,44,45]. Ablation rates 4 to 10 times higher than the best values for single pulses were achieved. Lasers used in GHz burst ablation typically have burst repetition rates of about 100 kHz [44,46]. For 100 kHz, the shifted burst method has only the advantage of continual scanning and would have only a 23% laser usage efficiency for the present texture geometry. The scanning speed would be only 0.7 m/s. On the other hand, the shifted path method combined with a fast galvanometer scanning head using a scanning speed of 20 m/s would use 99% of the laser time. That could lead to remarkably effective ablation rates and processing speeds without any heat accumulation and shielding. For example, for a burst energy of 500 µJ, it would mean 50 W of the average laser power would be used with a 99% efficiency, with a relatively simple scanning system.

The average laser power needed for the texturing methods ranged from 0.2 to 12.6 W (Table 2). The maximum is for the shifted burst method, which delivers the highest processing speed. The minimum is for the classic path method. These average power ratings are needed for running the methods at their maximum speed in this experiment. Effective average laser powers are obtained through a multiplication of the needed power by the laser usage efficiency. The effective laser powers are much lower (0.1–2.9 W). The lowest is for the classic hatch method, which offers the lowest processing speed. The ratio of the processing speed to the average laser power yields the resulting processing efficiency. The effective processing efficiencies are similar for all methods (50–69 mm^2^/(min·W)). This result was expected because the processes are similar and operate identical laser parameters. A point of interest here is the comparison of the needed processing efficiencies. The best is the shifted path method (64 mm^2^/(min·W)), followed by the classic path method. The worst is the classic hatch method, with an efficiency 10 times lower (6 mm^2^/(min·W)). The shifted burst method does not do well in this comparison either (14 mm^2^/(min·W)). Therefore, the shifted burst method is the fastest in terms of the processing speed (for the present experimental system), but the processing efficiency is not the highest.

The prediction obtained from the software suggests the existence of a linear dependence between the processing speed and scanning speed for the shifted method, and the limitation by the scanning speed was not observed. Therefore, presuming a continuation of the linear dependence and using a fast galvanometer scanning head with a scanning speed of 50 m/s (e.g., excelliSCAN), the processing speed would be 175 mm^2^/min for the path variant and 912 mm^2^/min for the burst variant. Presuming the use of a polygon scanning head with a 1 km/s scanning speed and a 71% time efficiency [47], the processing speed would be 2500 mm^2^/min for the path variant and 12,900 mm^2^/min for the burst variant. Such a laser process would require a 5 MHz repetition rate and a 55 W average laser power in the path variant and a 143 MHz repetition rate and a 1570 W average laser power in the burst variant. The burst variant with the mentioned parameters has not been solved yet (the high repetition rate and high laser power), but the path variant is already feasible. Such processing speeds would be much closer to the wide industrial applicability of laser surface texturing for functional applications and would enable an opening of new markets to this technology.

Further improvements are possible by combining the shifted LST method with other methods, such as multispot processing. A combination with the multispot approach can have a linear set of laser spots used in equidistant positions, with the spot distance set to the distance of objects in the texture, or its multiple. The scanning would be then done in a direction perpendicular to the line of the spots. The scanning can be done either by a galvanometer or polygon scanning head. In this way, a raster would be created by a single scan (or several scans); a small shift could be used to render the next raster. Both the shifted path and burst variants would be possible. Using this combination, even higher processing speeds can be achieved at the same texture accuracy.

## 5. Conclusions

In this work, the performance and accuracy of the shifted laser surface texturing method was verified. This method eliminates or significantly decreases the limiting factors of the current methods. Tests of the laser surface texturing of circular dimples (diameter 80 µm, depth 6.5 µm, distance 200 µm) on a stainless-steel surface were performed by two classic methods and the shifted method in two variants using a picosecond laser. The accuracy of the produced dimples was determined by the measurement of their size and circularity on high-contrast images of laser-ablated LabIR paint on the sample and by a depth profile measurement on the full texture using a contact surface profiler. The classic path method performed well (deviation < 5%) up to a scanning speed of 0.15 m/s, with a processing speed of 12.6 mm^2^/min. The classic hatch method had a limit at 0.7 m/s, with a processing speed of 3.4 mm^2^/min. Deviations > 15% were found at higher speeds (≥0.6 and ≥1 m/s). For the shifted method, no precision limit was found within the maximum speed of the present experimental system of 8 m/s for both variants. The depth profiles produced by the different methods at their maximal speeds showed similar deviations: 6% to 10%.

In the shifted path variant, the processing speed of 28 mm^2^/min was achieved and in the shifted burst variant, the value was at 146 mm^2^/min. This speed is 11 times higher than the result of the classic path method. The performance of the shifted method is dependent on the size of the textured area in the direction of the laser scanning and is more suitable for bigger sizes. The shifted burst method was identified as the best in the processing speed; the shifted path method showed the best processing efficiency per needed laser power (64 mm^2^/(min·W)). The processing speed is expected to grow linearly with the laser scanning speed for the shifted method. For a polygon scanner at 1 km/s and a 55 W picosecond laser, a processing speed of 2500 mm^2^/min is predicted to be attained (and even higher for femtosecond lasers). This speed is 200 times higher than the result of the best classical method and should be achieved with the same precision of the resulting texture.

The heat accumulation temperature increase was calculated for all methods. The maximum was found for the shifted burst method (320 K, 12 W laser power) and the minimum was found for the shifted path method (3 K, 0.4 W laser power). This suggests that also temperature-sensitive materials can be ablated at a high speed using the shifted path method and high average power lasers.

The laser usage efficiency of the different methods was compared. Values from 12% to 99% were obtained. The shifted path method (with a 99% usage efficiency) is very promising. Its combination with GHz burst machining was highlighted. A combination with the multibeam approach was also proposed.

## 6. Patents

Kucera, M.; Moskal, D.; Martan, J. Method of laser beam writing with shifted laser surface texturing. Patent application number PCT/IB2015/000807, WO2016189344. *World Intellect. Prop. Organ.* 2015; United States Patent 10160229, 2018.

## Figures and Tables

**Table 1:** Ablation rate and heat accumulation for different texturing methods.

Texturing Method	Scanning Speed (m/s)	Repetition Frequency (kHz)	Number of Layers	Pulses Per Layer	Ablation Rate (μm^3^/pulse) (μm^3^/μJ)		Heat Accumulation Δ*T* (K)
Classic Path	0.15	21	45	56	7.7	0.70	45
Shifted Path	8	40	60	54	7.6	0.69	3
Classic Hatch	0.7	100	85	42–50	5.6–4.7	0.51–0.43	96
Shifted Burst	8	1143	90	39–47	4.8–4.0	0.44–0.36	320

**Table 2:** Efficiency and processing speed for different texturing methods on a large processing area (2.4 × 170 mm^2^). Processing times and speeds predicted by software.

Texturing Method	Jump Delay Time (s)	Processing Time (s)	Jump Time Efficiency	Geometrical Efficiency	Laser Usage Efficiency	Average Laser Power (W)		Processing Speed (mm^2^/min)	Processing Efficiency (mm^2^/min/W)	
						Needed	Effective		Needed	Effective
Classic Path	413	1943	79%	99%	78%	0.24	0.18	12.6	53.5	68.6
Shifted Path	10	874	99%	100%	99%	0.44	0.43	28.0	63.6	64.4
Classic Hatch	1821	3600	49%	25%	12%	1.10	0.14	6.8	6.2	50.3
Shifted Burst	2	138	99%	24%	23%	12.57	2.91	177.0	14.1	60.8

## Supplementary Materials

The following are available online at https://www.mdpi.com/2072-666X/11/5/520/s1, Figure S1: SEM image of circular columns produced on tungsten surface by shifted burst method, Figure S2: SEM image of inclined circular columns produced on tungsten surface by shifted burst method, Figure S3: SEM image of donut holes produced by shifted path method on Al_2_O_3_ surface.
