# Supplementary material for: Performance and Accuracy of the Shifted Laser Surface Texturing Method
Source: Micromachines (Basel). 2020 May 20;11(5):520. doi: 10.3390/mi11050520 (PMC7281074; doi:10.3390/mi11050520)
Supplement: Supplementary file 1 [file micromachines-11-00520-s001.pdf]

micromachines

MDPI

Supplemental Materials

Performance and Accuracy of the Shifted Laser Surface Texturing Method

Jiří Martan *, Denys Moskal, Ladislav Smeták and Milan Honner

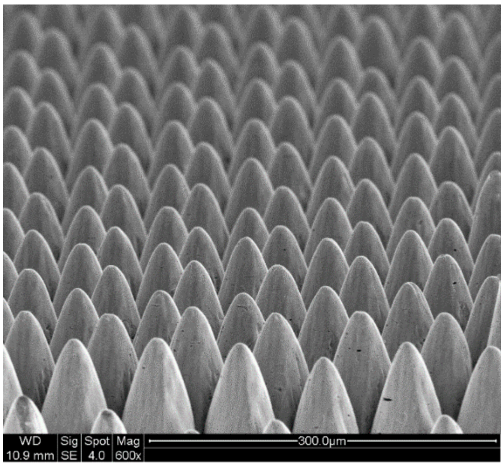

Figure S1. SEM image of circular columns produced on tungsten surface by shifted burst method. Laser beam scanning speed was 8 m/s, pulse energy 10 µJ, wavelength 532 nm, pulse duration 10 ps, spot size 23 µm, internal laser frequency (intra-burst) 1 MHz. External trigger frequency of the bursts was 112.6 kHz, gate opening time interval 5.1 ÷ 8.9 µs, distance between raster lines 63 µm, shifting vector length of sLST 10 µm.

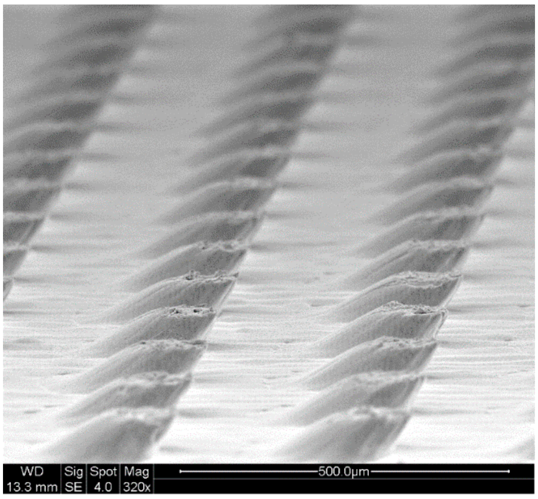

Figure S2. SEM image of inclined circular columns produced on tungsten surface by shifted burst method. Laser beam scanning speed was 8 m/s, pulse energy 10 µJ, wavelength 532 nm, pulse duration 10 ps, spot size 23 µm, internal laser frequency (intra-burst) 1 MHz. External trigger frequency of the bursts was 68.4 kHz, gate opening time interval 8.1 ÷ 14.6 µs, distance between raster lines 110 µm, shifting vector length of sLST 10 µm. The surface texturing was applied on sample surface with inclination of 45 degrees.

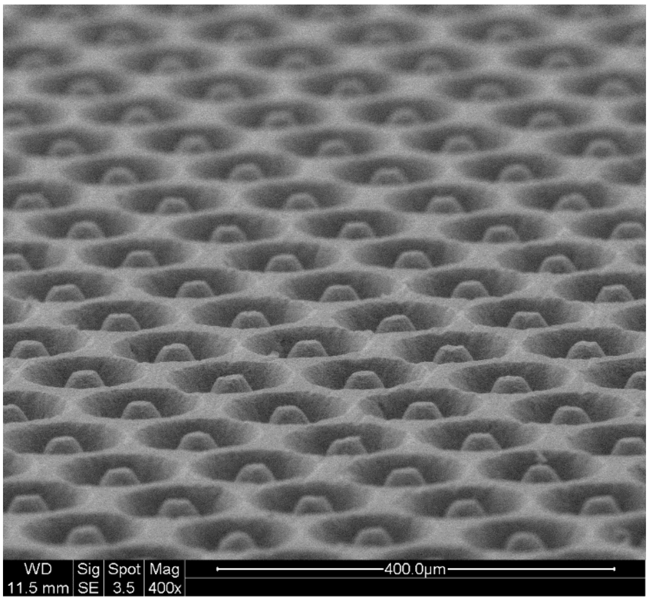

Figure S3. SEM image of donut holes produced by shifted path method on Al_2O_3 surface. The shifting trajectory was in the form of concentric circles (radiuses 21 µm and 56 µm). Laser beam scanning speed was 3 m/s, pulse energy 40 µJ, wavelength 532 nm, pulse duration 10 ps, spot size 23 µm, internal laser frequency 303 kHz. External trigger frequency in the shifted path method was 20 kHz, gate opening time interval 6.4 µs, distance between raster lines 120 µm, shifting vector length of sLST 5 µm.

© 2020 by the authors. Licensee MDPI, Basel, Switzerland. This article is an open access article distributed under the terms and conditions of the Creative Commons Attribution (CC BY) license (http://creativecommons.org/licenses/by/4.0/).
